# A Highly Sensitive and Selective Optical Sensor for the On-Line Detection of Cesium in Water

**DOI:** 10.3390/s23187826

**Published:** 2023-09-12

**Authors:** Alexis Depauw, Laura Jonusauskaite, Rasta Ghasemi, Jean-Pierre Lefevre, C. Mongin, Valérie Génot, Jacques Delaire, Isabelle Leray

**Affiliations:** 1 CNRS, Photophysique et Photochimie Supramoléculaires et Macromoléculaires, ENS Paris-Saclay, Université Paris-Saclay, 4 Avenue des Sciences, 91190 Gif-sur-Yvette, France; alexis.depauw@crimesciencetechnology.com (A.D.); laura.jonusauskaite@gmail.com (L.J.); cedric.mongin@ens-paris-saclay.fr (C.M.); valerie.genot@universite-paris-saclay.fr (V.G.); jacdelaire@orange.fr (J.D.); 2 Institut d’Alembert—FR 3242, ENS Paris-Saclay, 4 Avenue des Sciences, 91190 Gif-sur-Yvette, France; rasta.ghasemi@ens-paris-saclay.fr

**Keywords:** cesium, fluorescent molecular probe, microfluidic, sensor

## Abstract

In this study, we have undertaken the development of two fluorescent sensors based on calixarene compounds for the purpose of detecting cesium in water. By introducing the sulfonate functional groups, we have considerably improved the water solubility of sensors, enabling complete dissolution of products in aqueous media and direct analysis of polluted water samples. Through rigorous experiments, we have demonstrated that the complexation of Cs^+^ ions with sensors **1** and **2** in water leads to a remarkable enhancement of fluorescence. This fluorescence enhancement serves as a reliable indication of cesium presence and allows for sensitive detection. To further advance the practical application of our sensors, we have successfully integrated calixarene sensors **1** and **2** into a microfluidic sensor chip. This integration has enabled real-time, on-line measurements and has resulted in the development of a portable detection device capable of detecting cesium ions in water samples at parts per billion (ppb) levels. This device holds great promise for environmental monitoring and assessment, providing a convenient and efficient solution for cesium detection. Our work represents a significant advancement in the field of cesium detection, displaying the efficacy of calixarene-based fluorescent sensors and their integration into microfluidic systems. The enhanced water solubility, fluorescence response, and portability of our detection device offers tremendous potential for applications in environmental monitoring, water quality assessment, and emergency response scenarios where rapid and accurate cesium detection is crucial.

## 1. Introduction

Cesium, although relatively scarce on Earth with an abundance ranking of 45th and a concentration of 3 ppm in the Earth’s crust [1], exhibits a remarkable array of properties that render it captivating for a wide range of scientific applications. These properties include its electropositive nature, ductility as metal, large atomic radius, and high alkalinity. Notably, cesium’s applications encompass the efficient conversion of solar energy in perovskite compounds [2], the improvement of timing accuracy in atomic clocks [3], or even the treatment of cancers within the field of medicine [4].

The radioactive isotopes ^134^Cs (t_1/2_ = 2.1 years) and ^137^Cs (t_1/2_ = 30 years), generated through uranium, thorium, or polonium fission, represent highly toxic substances in terms of both biochemical and radioactive properties, and they constitute nuclear waste. The Fukushima nuclear power plant accident, triggered by the Tohoku tsunami in March 2011, resulted in a substantial release of radioactive materials, including cesium, into the environment. This event elevated concerns regarding cesium contamination in the anthroposphere. Cesium’s biochemical toxicity stems from its ability to replace potassium in muscles and red blood cells, potentially leading to cardiac and carcinogenic diseases [5]. Furthermore, cesium serves as a chemical tracer in thermal reactors, such as those in nuclear power plants [6]. Consequently, the detection of trace amounts of cesium has garnered significant interest.

The quantification of cesium levels can be achieved through various methods, including atomic absorption spectroscopy [7], inductively coupled plasma mass spectrometry [8], and radioanalysis [9]. However, these techniques necessitate sophisticated and costly equipment. Consequently, the development of portable devices has become highly appealing in terms of cost-effectiveness and ease of use. Promising approaches involve leveraging techniques such as electrochemistry [10], colorimetric detection utilizing functionalized gold nanoparticles and Prussian blue [11], on-chip microplasma [12], and field-effect transistors (EG-FET) [13]. Notably, a recent study has demonstrated the utilization of fluorescent CsPbBr3 perovskite immobilized in an ionic liquid membrane for low-cost sensing purposes [14].

In this particular context, the utilization of fluorescent sensors [15,16] offers numerous advantages in terms of sensitivity, selectivity, and response time. In the detection of Cs^+^ ions, multiple fluorescent molecular probes have been documented in the scientific literature [17,18,19,20,21] with the calixarene biscrown ether [22,23,24,25,26,27,28] being identified as the most efficient. Previously, we presented the synthesis, binding characteristics, and photophysical properties of a calix [4]arene-bis(crown-6-ether) incorporating a dioxycoumarin fluorophore within both crowns [29]. Additionally, a water-soluble analogue, containing sulfonate groups, has demonstrated intriguing complexing properties for cesium, exhibiting exceptional selectivity toward Li^+^, Mg^2+^, Ca^2+^, and Na^+^ ions [30]. These compounds are also K^+^ selective when the ratio of stability constants is greater than 500. Expanding upon this investigation, we have further integrated conjugation-extended coumarin units into the calix [4]arene framework, which exhibit a significantly larger Stokes shift when compared to the conventional coumarin fluorophore [14].

In our pursuit of fluorescent sensor development, various configurations have been explored [31,32,33,34,35,36]. Our long-standing approach involves combining a specific molecular fluorescent sensor with a microfluidic device, which has yielded successful results in previous studies targeting heavy metal detection (lead [37], mercury [38]). These achievements were accomplished using microfluidic chips that were efficiently filled with both the analyte and fluorescent molecular sensors via a syringe pump. In the previous case, since our fluorescent probes were not soluble in water, we have performed the measurements by using an organo-aqueous solvent, typically a mixture of acetonitrile and water. 

**Figure 1:**
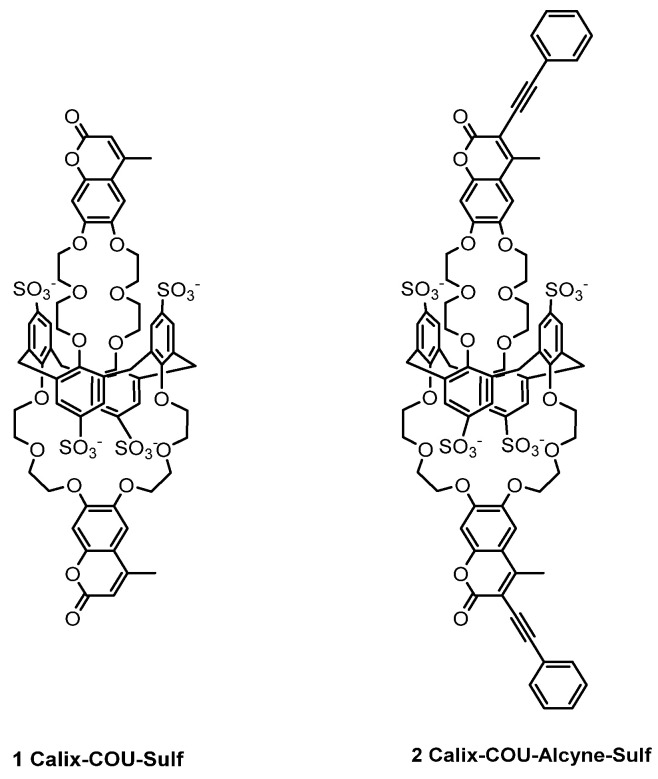
Chemical structure of molecular probes **1** and **2**.

Continuing our ongoing program aimed at continuous cesium detection in water, our current focus is on the development of a novel microfluidic device for on-line cesium detection. To achieve this, we have employed the calixarene-based fluorescent sensors **1** and **2** (Figure 1). In this manuscript, we present the photophysical properties of these fluorescent sensors alongside the details of our microfluidic setup. Furthermore, we provide a comprehensive evaluation of their sensing performance specifically tailored for cesium detection.

By integrating the calixarene-based fluorescent sensors into our microfluidic device, we aim to establish a robust and efficient system for real-time, on-line cesium detection in water. This approach not only offers enhanced sensitivity and selectivity but also enables continuous monitoring, overcoming the limitations of conventional detection methods. The presented results highlight the promising potential of our integrated system for applications in environmental monitoring, water quality assessment, and other relevant fields requiring continuous cesium detection capabilities.

## 2. Materials and Methods

### 2.1. Chemicals and Salts

The calixarene-based fluorescent sensor 1 was synthesized according to the previous procedure [30] by the chlorosulfonylation of a calixarene coumarin crown compound followed by hydrolysis of the chlorosulfonyl groups.

**NMR ^1^H** (300 MHz, (D_2_O)) 2.45 (6H, m, CH_3_), 3.15–3.35 (8H, m), 3.60–3.75 (8H, m), 3.75–3.85 (4H, m), 3.88–3.96 (4H, m), 4.07 (8H, br s), 4.11–4.27 (8H, m), 6.23 (2H, s), 7.03 (2H, s), 7.29 (2H, s), 7.60 (8H, s); 

**NMR ^13^C** (75 MHz, D_2_O) 18.9 (CH_3_), 37.7 (Ar–CH_2_–Ar); 69.1, 69.5, 69.7, 69.8, 70.4, 70.9, 71.0 (O–CH_2_); 103.0, 111.3 (CH_ar_); 113.3 (Cq), 114.5, 127.5 (CH_ar_); 134.6, 134.7, 138.3, 138.4, 146.0, 150.4, 154.1, 157.5, 159.2, 162.2, 165.9 (Cq);

**MS***m*/*z* (ESI_2_): 724.9 ([M^–2^Na]22), 475.6 ([M^–3^Na]32), 351.0 ([M^–4^Na]42).

The synthesis of the compound **2** was performed according to [39] with the iodination of Calix-COU-Sulf 1 with NIS and TFA in a 1:1 (*v/v*) mixture of acetonitrile and methanol followed by Sonogashira cross-coupling reactions between the iodo calixarene and phenylacethylene compound in the presence of CuI and Pd(PPh_3_)_4_.

**NMR ^1^H** (400 MHz, MeOD): δ (ppm) 7.63 (s, ^8^H_calix_), 7.59–7.57 (m, 4H, CH_ar_), 7.39–7.38 (m, 6H, CH_ar_), 7.23 (s, 2H, CH_coumarine_), 6.99 (s, 2H, CH_coumarine_), 4.30–4.23 (m, 16H, OCH_2_), 3.92–3.86 (m, 24H, CH_2_ + OCH_2_), 2.76 (s, 6H, CH_3_).

**NMR ^13^C-DEPT-135** (100 MHz, MeOD): δ (ppm) 131.34 (CH_ar_), 129.29 (CH_ar_), 128.69 (CH_ar_), 126.79 (CH_ar_), 111.51 (CH_ar_), 102.15 (CH_ar_), 70.74 (OCH_2_), 70.53 (^2^OCH_2_), 70.31 (OCH_2_), 69.97 (OCH_2_), 69.87 (OCH_2_), 69.31 (OCH_2_), 69.26 (OCH_2_), 37.17 (CH_2_), 18.83 (CH_3_). 

**HMRS (TOF MS ES+):** found: [M^−3^Na]^3−^ = 542.4223; Calc. for C_80_H_68_Na_4_O_28_S_4_ [M^−2^Na]^2−^ = 542.4225.

The cesium, potassium, lithium, calcium, and magnesium salts used in our study and obtained from Sigma-Aldrich, Saint Quentin Fallavier, France were of the highest quality ‘99.99% metal basis’ available and were vacuum dried over P_2_O_5_ before use.

### 2.2. Spectroscopic Measurements

The UV/V absorption spectra was acquired in a 1 cm optical length quartz cuvette using a double-beam Varian Cary 5000 spectrophotometer (Les Ulis, France) equipped with deuterated/halogen lamps. Corrected emission spectra was obtained utilizing a Jobin–Yvon Spex FluoroMax (Palaiseau, France) spectrofluorometer with a xenon light source. A right-angle configuration was used. To determine the fluorescence quantum yields, quinine sulfate dihydrate in 0.5 N sulfuric acid was employed as a standard with a known quantum yield (Φ_F_ = 0.546) [40]. It is important to note that the estimated experimental error associated with these measurements was less than 10%. During emission measurements, strict precautions were taken to maintain accuracy. The absorbance at the excitation wavelength was deliberately kept below 0.1, ensuring that any potential interference or self-absorption effects were minimized. Titration experiments were performed by adding a cesium solution to the ligand **1** and **2**. The complexation constants were determined via global analysis of the evolution of all absorption and/or emission spectra using the Specfit Global Analysis System V3.0 for the 32-bit Windows system. This software uses singular value decomposition and non-linear regression modelling via the Levenberg–Marquardt method.

### 2.3. Microfluidic Device

**Figure 2:**
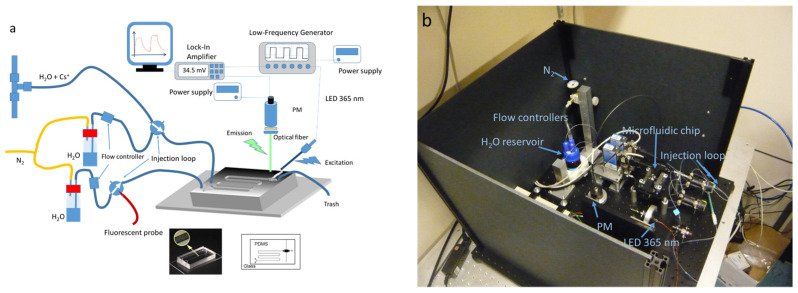
(**a**) Scheme of the microfluidic device for on-line detection of Cs+; (**b**) photograph of the complete detection system.

The device depicted in Figure 2 represents a significant component of our experimental setup. It comprised a Y-shaped microchannel fabricated from polydimethylsiloxane (PDMS) obtained from Dow Corning, which was affixed to a glass substrate (standard microscope slide measuring 50 mm long × 25 mm wide) [37]. The microchannel dimension was typically 200 μm wide and 100 μm high. To facilitate effective mixing of the fluorescent probe solution and cesium solution, a passive mixer with ridges 60 μm wide and 30 μm high, analogous to that described by Stroock et al. [41], was incorporated into the device. The microfluidic circuit was continuously supplied with pressurized water (0.4 bar) from a dedicated tank, utilizing capillaries with an inner diameter of 250 µm. Each channel of the circuit was equipped with a flow regulator (Bronkhorst μ-FLOW L01) to precisely control the flow rates at the circuit inlets (0.65 mL/h). Injection of the probe and cesium solutions was achieved using valves equipped with 750 µm inner diameter injection loops (Upchurch V451). The concentration of the probes **1** and **2** in water were fixed at 4 µM.

To excite the fluorescent probes, an SMA connectorized LED emitting at a wavelength of 365 nm (UVLED365-10E, Optoprim, Vanves, France) was employed. The excitation light was filtered using an optical filter with a 10 nm bandwidth centered at 370 nm (Semrock FF01-370/10-25, Optoprim, Vanves, France). A fused silica ball lens was utilized to focus the light beam onto an optical fiber. The optical fiber, connected to the LED via an SMA optical connector, was inserted into the PDMS circuit in close proximity to the microfluidic channel. To enable heterodyne detection of the fluorescence, the LED was supplied with square signals at a frequency of 77 Hz by a low-frequency generator [37].

Fluorescent light was collected using a Thorlabs FT100UMT fiber (core size 1000 µm, bandwidth 300–1200 nm, numerical aperture 0.39) and directed through a high-pass filter (Semrock FF01-550/200-25, Optoprim, Vanves, France) positioned at the entrance of the photomultiplier tube (Hamamatsu H10722-210, Hamamatsu, Massy, France). The voltage signal generated was then routed to a lock-in amplifier (Signal Recovery 7265 DSP, Hi-Tech DETECTION SYSTEMS, Massy, France) and acquired on a computer via an RS232 bus. The recorded voltage signal typically reached approximately 100 millivolts, considerably exceeding the signal-to-noise ratio of one, ensuring reliable and accurate measurements.

## 3. Results and Discussion

### 3.1. Complexing Properties with Cesium

**Figure 3:**
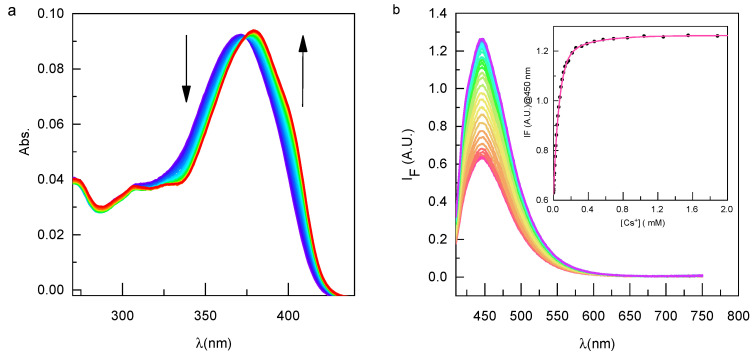
Progressive changes in the absorption (**a**) and emission (**b**) spectra of compound **2** (2.4 μM) upon the introduction of cesium acetate in water (0 to 1.2 mM of Cs^+^ from red to blue). The inset of (**b**) displays a calibration curve, highlighting the correlation between cesium concentration and fluorescence intensity (λ_exc_ = 405 nm, λ_em_ = 450 nm).

Prior to fabricating the microfluidic sensor, an assessment was conducted to examine the complexing properties of compound **2** and a comparative analysis was conducted with the results obtained for compound **1** (Figure 3). Similar to compound **1**, the introduction of cesium ions led to a bathochromic shift of 8 nm in the absorption spectra, which manifested as the emergence of an isobestic point observed at 374 nm. Such effect can be attributed to the interaction of the bound cation with the oxygen of the coumarin. In comparison to Calix-COU-Sulf 1, the absorbance exhibited only a marginal increase. This observation can be attributed to the screening effect stemming from the presence of sulfonate functional groups [30].

Through meticulous analysis of the absorption spectra of compound **2**, utilizing the SPECFIT program, it was discerned that successive formation of 1:1 and 2:1 (metal to ligand) complexes with cesium ion occurred. This observation aligns with the presence of two binding crown-ether complexation sites within **2**. The stability constants K_11_ and K_12_ for these complexes are determined by employing the following equations:

(1) $$M+L⇌ML\;K_{11}=\frac{\left[ML\right]}{\left[M\right][L]}$$

(2) $$M+ML⇌M_{2}L\;K_{21}=\frac{\left[M_{2}L\right]}{\left[ML\right][M]}.$$

While the overall binding constant β_21_ can be defined as

(3) $$2M+L⇌M_{2}L\;\beta_{21}=\frac{\left[M_{2}L\right]}{{\left[M\right]}^{2}\left[L\right]}.$$

**Figure 4:**
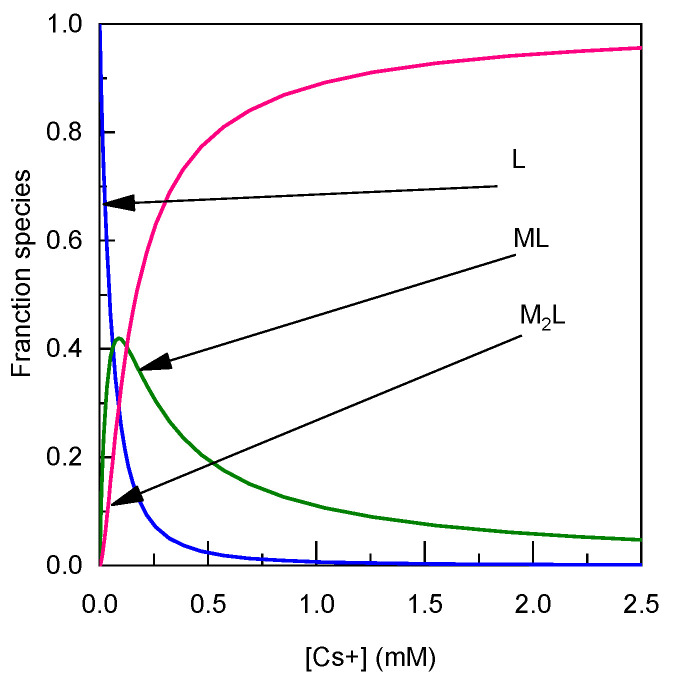
Calculated speciation diagram of 2 (2.4 µM) and its 1:1 and 2:1 complexes with Cs^+^ from the titration curve and the binding constant extracted from data presented in Figure 2 using Equations (1) and (2).

The stability constants of the complexes of Cs^+^ with 2 were determined to be Log K_11_ = 4.22 ± 0.02 and Log K_21_ = 3.91 ± 0.07 (Table 1). These values are consistent with those observed for compound **1** and other calixarene-based cesium fluorescent sensors [25], indicating comparable binding capabilities. The ratio of K_21_/K_11_ is 0.49, indicating equivalent complexation sites. Such an effect is observed because of the participation of the sulfonate group on the complexation. Figure 4 presents the speciation diagrams depicting the formation of species resulting from the assay. As shown in Figure 4, at low cation concentrations, only complex 1 to 1 is formed, while complex 2 to 1 is formed at higher concentrations. In order to form 90% of the complex M_2_L, the concentration of Cs^+^ has to be 2 mM.

It should be noted that both compound **1** and **2** exhibits high fluorescence efficiency, and the fluorescence quantum yield is around 0.4 for both compounds. Since these compounds have a high molar extinction coefficient, the brightness defined as Ф_F_ × ε is high for **1** and **2**, which is a significant advantage for sensor applications.

Similar to compound **1**, the addition of cesium induces an increase in fluorescence intensity without causing a shift in the spectra. The fluorescence enhancement exhibits linearity within the concentration range of 0 to 50 µM cesium, with an overall twofold increase. Notably, the fluorescence response ΔI_F_ of compound **2** is less pronounced compared to compound **1**. Detailed data are reported in Table 1.

### 3.2. Microfluidic Device

The implementation of microfluidics for the fluorescence-based detection of heavy metals in water offers significant advantages, including low sensor molecule consumption, portability of the device, and high sensitivity and selectivity. In our previous approaches, both the analyte and sensor solutions were continuously flowing and mixing within the system. The complexation of the sensor with the analyte led to a noticeable change in the fluorescent signal, which was detected at the flow endpoint. However, this configuration relied on syringe pumps, requiring manual manipulation to change the solutions in the syringes and increase the risk of fluorescent sensor adsorption onto the microfluidic system walls.

In our ongoing program focused on the continuous detection of cesium in water, we are exploring the use of an injection loop configuration. This approach ensures a continuous flow by applying pressure upstream and utilizing a regulated flow meter. Since our fluorescent probes **1** and **2** are completely soluble in water, we can use them without cosolvents (acetonitrile, methanol). Thus, the analyte and sensor solutions are injected into the system through an injection loop and pushed at a controlled flow rate through the microdevice (0.5–1 mL/h for each solution). Notably, the microdevice is solely traversed by water flowing at the same rate, enhancing the system’s longevity. The setup is depicted in Figure 2.

To supply pressure to the microfluidic chip, we employ a configuration that offers several advantages. Two capillaries are immersed in a tank of ultra-pure water, subjected to an air pressure of 0.4 bar, enabling the precise delivery of water to the two input channels of the microsystem. The flow rates in each channel are carefully regulated using flow regulators, ensuring optimal control.

Additionally, each channel incorporates a two-position valve, commonly utilized in chromatography, facilitating the injection of the probe **1** or **2** and analyte solutions. This setup has notable benefits, including limited contact of the chemicals with the PDMS channels solely during the measurement process, effectively preventing adsorption issues on the microchannel walls. Consequently, the chip remains consistently clean, significantly extending its lifespan.

The measurement time is meticulously optimized by adjusting the length of the injection loop. This optimization strikes a balance between obtaining prompt results and acquiring sufficient statistical data, thereby enhancing result reliability and improving the signal-to-noise ratio.

Upon mixing with the passive mixer, the complexation of the ligand with the analyte occurs within the microchannel. The resulting fluorescence signal is then collected at its end of the microfluidic channel. Moreover, the solution is excited perpendicularly to the microchannel using an LED (365 nm), achieved through an optical fiber embedded in the PDMS. Finally, the emission is collected at a 90° angle through an optical fiber connected to a photomultiplier tube (PMT) with a high-pass filter.

**Figure 5:**
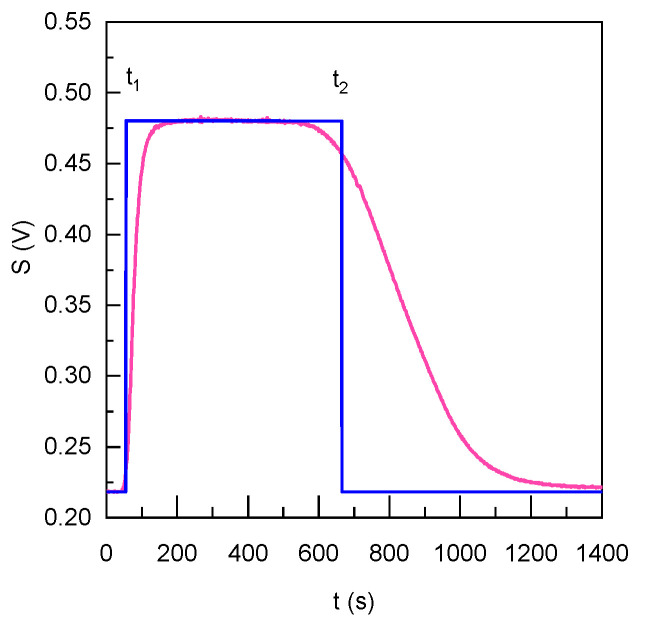
Fluorescence profile inside the microfluidic chip upon the addition of **2** (4 µM).

The signal obtained has a profile of an asymmetrical plateau whose level depends on the intensity of the measured fluorescence (see Figure 5). According to the diameter of the capillaries, the theoretical plateau is shown in blue in Figure 5 starting at t_1_ = 45 s and finishing at t_2_ = 45 + 610 s. The obtained signal rises for 150 s and reaches the plateau during 300 s. The signal then decreases slowly to reach the initial value of the noise due to the flow of pure water.

The shape and the duration of each sequence (stage) depend primarily on the geometry of the microfluidic channels and on the flow rates. The profiles are heavily caused by axial and radial dispersion phenomena. Indeed, the axial elongation of the flow due to the laminar Poiseuille flow coupled to the radial diffusion of the products due to their concentration gradient causes the Taylor dispersion phenomenon [42]. This phenomenon is important in cylindrical tubes whereas it becomes negligible in the area of PDMS microfluidic system because of the herringbone structures and the relatively large Peclet number.

**Figure 6:**
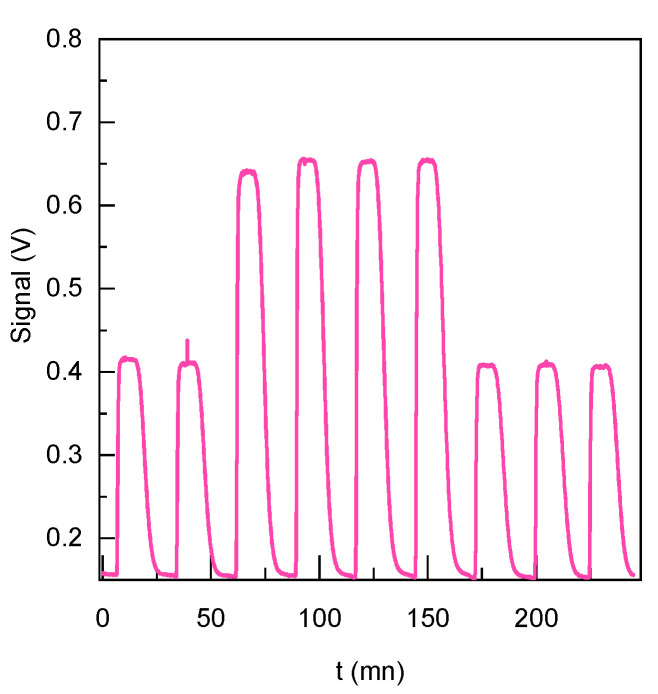
Sequential injection of compound 2 samples (1.84 µM). The sensor alone exhibits the low-level signal, while sensor + Cs^+^ represents the highest level (20 µM). This sequential injection approach demonstrates the system’s sensitivity and reversibility.

The stability of the system was comprehensively assessed, and the findings, illustrated in Figure 6, demonstrate its complete reversibility. The relative errors for the signal of the ligand **2** and the ligand **2** complexed with Cs^+^ (20 µM) is less than 0.8%. It is also interesting to note that, thanks to the lock-in detection, the signal-over-noise ratio is very good (S/N > 50). Remarkably, the system promptly returns to its initial level by simply adjusting the concentration of cesium. This reversible behavior highlights the robustness and reliability of the system, emphasizing its potential for the precise and controlled detection of cesium. 

**Figure 7:**
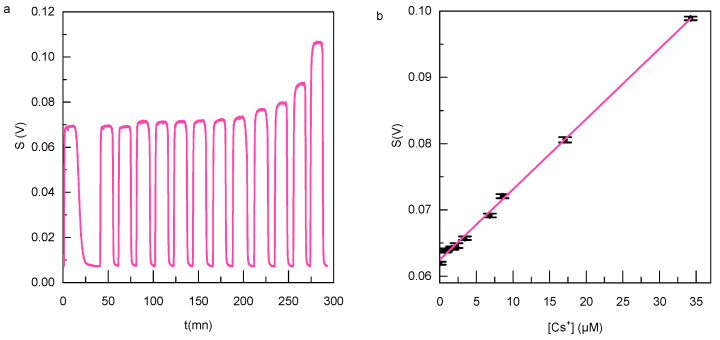
(**a**) Variation of the measured fluorescence intensity of **1** (4 µM) at the output of the microfluidic circuit for different concentrations of cesium acetate; (**b**) calibration curve for the determination of Cs^+^ ions by **1** (4 µM) in H_2_O. The error bar represents the standard deviation.

In order to detect cesium, the detection process involved the utilization of probe sensors **1** and **2**. The concentration of the probe solution was carefully selected based on the targeted cesium content for detection. The injection loops’ “probe” and “cesium” channels (see Figure 7) were supplied with either the probe solution and cesium solutions of known concentrations to establish a calibration curve or with the probe solution and the analyte effluent obtained from the bypass circuit. This approach facilitated the assessment of fluorescence changes upon the addition of various cesium concentrations. As previously observed in the cuvette, a significant enhancement of the fluorescence emission is observed upon complexation with cesium.

The sensitivity of compounds **1** and **2** towards Cs^+^ ions was assessed following the established standard protocol as described in previous studies [43]. The detection limit was determined based on the generated calibration curve (Figure 7b) using Equation (1), as outlined below.

DL=K×σ/S

where *K* = 3 is the detection factor for the detection limit, *σ* is the standard deviation of the blank, and *S* is the slope of the calibration curve.

The standard deviation of the blank has been calculated with sufficient data measurements (100 points) 

In an aqueous solution, the detection limit for compound **1** was determined to be 153 nM (21 ppb), while for compound **2** it was found to be 205 nM (28 ppb). Notably, the detection limit achieved in our microfluidic circuit was lower than that in conventional spectrofluorimeters, thanks to the improved signal-to-noise ratio of our detection device. To the best of our knowledge, this water-soluble sensor stands among the most sensitive sensors reported in the literature [10].

## 4. Conclusions

In this study, we have successfully designed and developed a sensor for cesium ion detection. The sensor utilizes molecular fluorescent probes based on calixarene crowns functionalized with coumarins, which exhibit high fluorescence intensity (Φ_F_ > 0.3). Upon complexation with cesium in water, these probes undergo significant changes in their photophysical properties, including absorption and fluorescence. Analysis of the assay curves revealed the formation of two complexes with stoichiometries of 1:1 and 2:1 (M:L), with high stability constants (>10^4^) accompanied by a remarkable enhancement in fluorescence. The detection limit has been found to be 600 nM for both compounds in the cuvette.

To enable practical applications, we developed a microfluidic device that incorporates these fluorescent probes and facilitates continuous feeding for analysis. The device demonstrated excellent sensitivity in detecting cesium in water, with detection limits of 153 nM for compound **1** and 205 nM for compound **2**. These results are highly promising and suggest the potential for integrating such sensors into portable devices, enabling rapid and cost-effective monitoring of Cs^+^ in water.

## Figures and Tables

**Table 1:** Photophysical and complexing properties of **1** and **2**.

	1	2
Log K_11_	4.10 ± 0.04	4.22 ± 0.02
Log K_21_	3.82 ± 0.05	3.91 ± 0.07
K_21_/K_11_	0.5	0.49
ε (M^−1^.cm^−1^)	21,000	37,500
Φ_F_	0.39	0.43
ΔI_F_ (λ_exc_)	2.5 (365 nm)	2 (405 nm)
LOD (µM)	0.6	0.6

## Data Availability

Not applicable.
